# Laparoscopic assisted anorectal pull through: Reformed techniques

**DOI:** 10.4103/0971-9261.59604

**Published:** 2009

**Authors:** Karthik S. Bhandary, V. Kumaran, G. Rajamani, S. Kannan, N. Venkatesa Mohan, R. Rangarajan, V. Muthulingam

**Affiliations:** Department of Pediatric Surgery, Coimbatore Medical College Hospital, Coimbatore, India

**Keywords:** Anorectal malformations, Laparoscopic assisted anorectal pull through, puborectalis muscle, pull through channel.

## Abstract

**Aim:**

To assess the modifications in the technique of laparoscopic assisted anorectal pull through (LAARP) practiced at our institute and analyze the post operative outcome and associated complications.

**Materials and Methods:**

A retrospective study from January 2001 to May 2009 analyzing LAARP for high anorectal malformations.

**Results:**

A total of 40 patients - 34 males and six females, in the age group of two months to six years were studied. Staged procedure was done in 39 patients; one child with recto vestibular fistula underwent single stage procedure. All the patients withstood surgery well. One patient required conversion due to problems in gaining enough length for the distal rectum in a patient with rectovesical fistula so colostomy was closed and re-located at a proximal splenic flexure. The complications were mucosal prolapse (six cases), anal stenosis (three), adhesive obstruction (two), distal rectal necrosis (one), and urethral diverticulum (one). The patients were followed up with clinical evaluation and continence scoring. The progress has been satisfactory and weight-gain is adequate.

**Conclusions:**

The advantages of the reformed techniques are as follows: Transcutaneous bladder stitch provides excellent visualization; traction over the fistula helps in dissection of the puborectalis, dividing the fistula without ligation is safe, railroading of Hegar's dilators over the suction canula creates adequate pull through channel, saves time and makes procedure simpler with reproducible comparable reports.

## INTRODUCTION

Georgeson *et al.*[1] described laparoscopically assisted anorectal pull through (LAARP) for high anorectal malformations to reduce the amount of posterior dissection required for accurate placement of the bowel into the muscle complex. At our institution, we have been performing LAARP since 2001. Over the period of years we have made technical modifications, which have made the procedure simpler, decreased the operative time and improved postoperative results. The present study is intended to discuss the technical modifications of LAARP as practiced at our institute and analyze the postoperative outcome and complications associated with our technique.

## MATERIALS AND METHODS

This is a retrospective and prospective study analyzing LAARP performed at the Department of Pediatric surgery, Coimbatore Medical College Hospital. Coimbatore from January 2001 to May 2009. A total of 40 patients had undergone LAARP, with 34 male and six female patients. The age group ranged from two months to six years. Staged procedure was done in 39 patients and primary pull through in one child of recto vestibular fistula of five months of age.

### Operative Technique

We prefer the three-stage repair i.e. neonatal colostomy followed by LAARP with stoma closure in third stage. After adequate bowel preparation, intravenous antibiotic cover was given an hour before surgery. Endotracheal general anesthesia with caudal block was given.

### (A) Ergonomics

**Figure 1:**
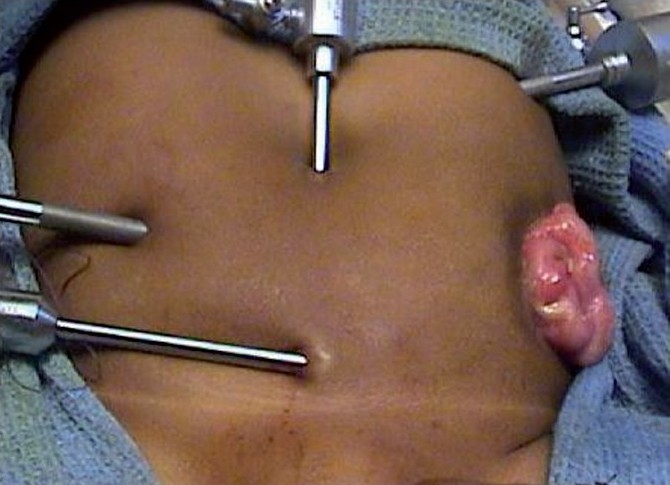
Port placement

The child was placed in the Trendelenburg position with legs widely spread (frog position) and tilt to the pelvis. The surgeon to the right and the assistant to the left stand at the head end while second surgeon (required at the later point in the operation) works from the foot end. Pneumoperitoneum is created with CO_2_ (8-12 mm of Hg) by the open method. The abdomen is accessed by three ports, one 5 mm umbilical port for 30° telescope and two 5mm accessory working ports one at right lumbar region and another at left hypochondrium. For comfort, an additional 5mm suprapubic port or a stay suture will help retraction of the bladder [Figure 1].

### (B) Rectal dissection

The urinary bladder is catheterized. Early in the dissection, a Hegar's dilator is placed in the mucous fistula to retract the rectum. The bladder, despite being decompressed, is retracted anteriorly by a transcutaneous bladder stitch. Dissection is commenced at the level of the peritoneal reflection. The terminal branches of the sigmoid and superior rectal arteries are divided to gain adequate length. The distal rectal peritoneal attachment is released with bipolar dissection and is continued anteriorly and laterally on the rectal wall taking care not to injure ureter and genital structures.

### (C) Fistula identification

Hook cautery is traded for bipolar scissors once the dissection reaches the bladder neck to avoid lateral damage to the pelvic nerves. Anterior dissection is stopped on identifying prostate / lower end of the uterus usually visualized as an indentation through the posterior vaginal fornix. The distal colon is now isolated to its fistulous connection.

**Figure 2:**
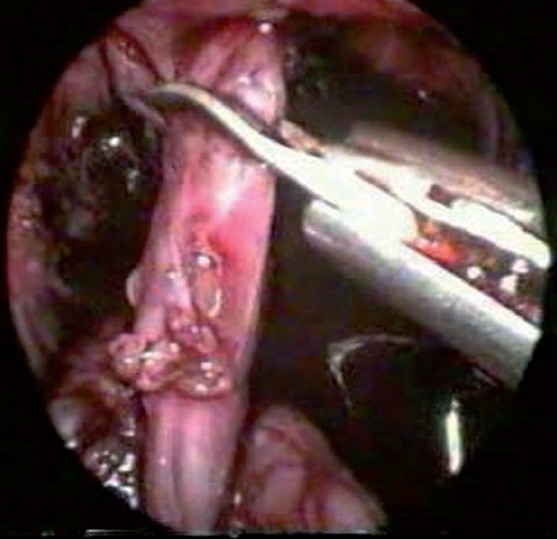
Fistula division

The higher the fistula, the easier the dissection; it stops at the point of coning or narrowing, which occurs before its fistulous communication. Puborectalis sling is identified before fistula division, traction on the fistula helps in midline dissection to create the pull through channel. A small cuff of fistulous tissue is left behind to prevent injury to the vaginal or urethral wall and injury to pelvic nerve plexus. Fistulous division [Figure 2] is done without ligation.[2]

### (D) Creating pull through canal

**Figure 3:**
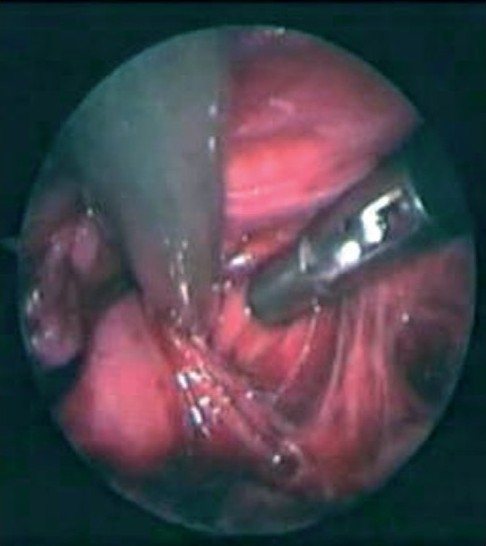
Pubococcygeus bellies

With cephalad traction on the fistula, both bellies of pubococcygeus can easily be identified [Figure 3] in relation to the urethra. The classic anatomic arrangement of the puborectails,[1] resembling a “sling–shot,” can often be appreciated. The contractility of the levator ani muscle and center of its two bellies is identified by the laparoscopic muscle stimulator.

Conventional diathermy in a low setting current can be used just as effectively as a laparoscopic muscle stimulator When there is insufficient muscle mass to clearly ascertain the pubococcygeus, the midline is identified based on the position of the distal end of the divided fistula and the urethra. Externally, the anal area of the perineum is mapped out using transcutaneous electro stimulation (muscle stimulator with 100-150 milliamps current). The area of maximal contraction and ventrocephalad elevation of the perineum is noted with simultaneous contraction of the puborectalis. The anterior and posterior limits of this anal area are marked and a 12mm vertical midline incision is made at the proposed anal orifice.

The intrasphincteric plane is dissected bluntly from below to the level of the levator sling using laparoscopic back light as guide from above. The dissected intrasphincteric plane is dilated with serial Hegar's dilators up to 10-12 mm size railroaded over the suction canula between the two bellies of the pubococcygeus muscle in the midline

### (E) Rectal pull through

A 10 mm trocar is inserted through dilated tract into peritoneal cavity. The divided rectal fistula is grasped using an endo-Babcock clamp and pulled onto the perineum through the newly created tract taking care not to twist the bowel. Anoplasty is done with 4-0 vicryl stitches. Finally the rectum is retracted cephalad and secured to the presacral fascia with 2-0 silk seromuscular sutures two on either side of the rectum. This retraction lengthens the skin lined anal canal.

### (F) Comparison with conventional technique [Table 1]

In Georgeson's procedure the baby is placed transversely at the end of the table so as to allow access on three sides by the operating team with spreading of legs during the perineal dissection. Our improvement on it is the Trendelenberg position with legs widely spread (frogs position / lithotomy), so that the position of the baby remains unchanged. In the conventional method, bladder is just decompressed but it still hangs hindering the visualization of the pelvis and levator. We place a transcutaneous bladder stitch to overcome this obstacle. Traction on the undivided fistula helps in defining the sling shot of puborectalis and blunt dissection in the center of it for the pull through channel. Fistula ligation as proposed was omitted as the outcome was similar with simple division only. Conventional diathermy in a low setting current can be used just as effectively as a laparoscopic muscle stimulator originally proposed by Georgeson. Instead of a low profile step, Verees needle with expanding sheath placed through proposed anal site for accurate creation of pull through channel under suction canula guidance blunt dissection is done from the perineum and Hegar's dilators up to 12 mm size are successively railroaded to dilate the pull through channel.

**Table 1:** Authors' modifications from Georgeson's procedure

Georgeson's Procedure	Modifications
Position – baby placed transversely at the end of the table with spreading of the legs during perineal dissection	Trendelenberg position with legs widely spread (frogs position / lithotomy), so that the position of the baby remains unchanged.
The bladder is just decompressed but it still hangs hindering the visualisation of the pelvis and levator.	We place a transcutaneous bladder stitch to overcome this obstacle
Fistula ligation is mandatory before division	Fistula ligation is omitted
Laparoscopic muscle stimulator is used for puborectalis muscle stimulation.	Conventional diathermy in low setting current can be used as effectively.
A low profile step Verees needle with expanding sheath placed through proposed anal site for accurate creation of pull through channel.	Under suction canula guidance blunt dissection is done from the perineum and Hegar's dilators up to 12 mm size are successively railroaded to dilate the pull through channel

## RESULTS

Thirty five cases have completed all three stages, five cases are awaiting colostomy closure and all are on regular follow-up. The age wise distribution, site of fistula, associated anomalies and complications in our patients are shown in Tables 2–5 respectively.

**Table 2:** Age Distribution

Age	No. of Cases	Percentage
1-6 Months	7	17.5
7-12 months	16	40
1-2 years	12	30
3-6 years	5	12.5

**Table 3:** Type of fistula.

Recto urethral	32	80
Recto vesical	3	7.5
Recto vaginal	2	5
Recto vestibular	3	7.5

**Table 4:** Associated anomalies

Associated anomalies	No of cases
Solitary Kidney	3
MCDK	3
Penile Duplication	1
ASD	2
PDA	2
Undescended Testis	4
Bifid Scrotum	1
Urethral Duplication	1
Hypospadias	1
Albinism	1
Down Syndrome	1
Polydactyly	1
Sacral Anomalies	1

**Table 5:** Complications

Complications	No of cases
Mucosal prolapse	5
Anal stenosis	2
Adhesive obstruction	2
Distal Rectal Necrosis	1
Urethral diverticulum	1

All the patients tolerated surgery well. One patient required conversion due to problem in gaining enough length for the distal rectum. This occurred in a patient with rectovesical fistula and in this case colostomy was closed and relocated at splenic flexure.

Mucosal prolapse is the commonest complication in our series which required mucosal trimming. This can be prevented by taking seromuscular sutures through rectum to presacral fascia while placing cephalad tension laparoscopically. Anal stenosis occurred in two cases and treated with posterior triangular anoplasty. Two patients developed adhesive intestinal obstruction which was managed conservatively in one patient and another patient required laparotomy and adhesiolysis. There was a peroperative spill of distal rectal contents particularly barium in these two patients.

Distal rectal necrosis of 1.5 cm of rectum near the neoanus occurred in one patient. Peroperatively distal vascular branches to the rectum were sacrificed for adequate mobilization. The child was treated with a revision pull through. Urethral diverticulum occurred in one patient, probably due to leaving behind a long stump of recto urethral fistula. The patient is asymptomatic on follow-up.

### 

#### Follow-up:

**Figure 4:**
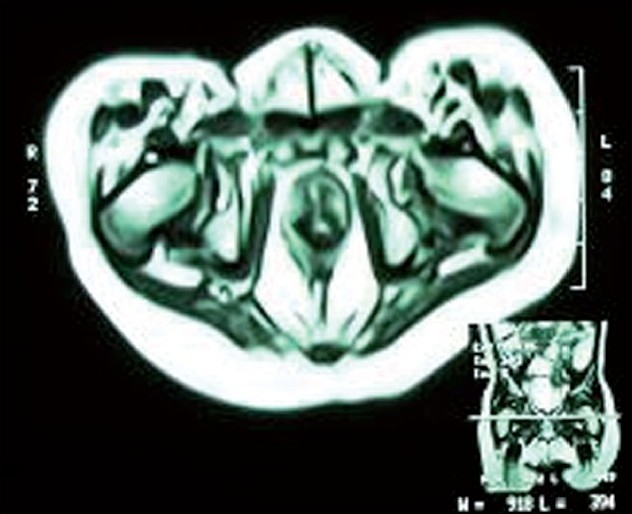
Postop MRI appearance of pelvis

In all children, the progress has been satisfactory and weight gain was adequate. All our patients pass formed stools two to three times a day and have symmetric anal contraction on stimulation. Anal ultra sonogram done in individuals showed a symmetric muscle complex on either side of pull through rectum. Distal loopogram done prior to colostomy closure has shown a good anterior angulation of rectum reflecting accurate placement of rectum within the puborectalis sling. CT scan of pelvis showed the neo-rectum placed in the center of levator sling and within the anal sphincter. Similarly MRI pelvis was done in selected affording patients who showed the neo-rectum placed in the center of levator sling and within the anal sphincter [Figure 4].

#### Postoperative continence:

Thirty two patients could be evaluated postoperatively according to international classification[3] for postoperative results as shown in Table 6. Twenty seven patients are beyond three years of age and their social continence is good.

**Table 6:** Postoperative continence.

Voluntary bowel movements	YES
(Feeling of urge, Capacity to verbalize, Hold the bowel movement)	
Soiling	GRADE 1 - 3/32
	Occasionally
	(once or twice per week)
	GRADE II - 1/32
	Every day, no social problem
	GRADE III - 1/32
	Constant, social problem
Constipation	GRADE 1 – Nil
	Manageable by changes in diet
	GRADE II - 2/32
	Requires laxative
	GRADE III - Nil
	Resistant to laxatives and diet changes

## DISCUSSION

Early in the 20^th^ century, an anorectal pull-through procedure was used for high lesions. As surgical techniques improved, endorectal pull through, abdominoperineal pull through and later the sacroabdominoperineal approach came into being. However, in many of these cases, identification and visualization of the levators and external sphincter muscle complex was not possible and the anorectum often was passed “blindly” into its final position.

Posterior Sagittal Anorectoplasty (PSARP), popularized by deVries and Pena,[4] revolutionized the management of infants with imperforate anus. This approach provided excellent visualization, protection of the urogenital structures,[3] the ability to mobilize the bowel sufficiently, and identification of the urinary fistula. Fecal continence,[5] improved using this approach but often is less than ideal in patients with high fistulae.[6–8]

In an attempt to improve on these results, Keith Georgeson's[1] LAARP uses fundamental concepts learned from decades of high ARM repair incorporating modern technologic advancements. An anatomic reconstruction of the ARM (such as that which results after PSARP) could be achieved with minimal surgical trauma to the continence mechanism (e.g., pelvic nerves and musculature),

Benefits of the procedure include lack of division of the muscle complex, no need for laparotomy, decreased pain to the patient, and potentially less perineal wound complications. Additional advantages include repair of associated defect at operation (i.e., hernia, identification and repair of cryptorchid testes), superior pelvic visualization not possible with open surgery, and anatomic placement of the pull -through bowel by identifying the central portion of the puborectalis from inside and the external anal sphincter from outside the patient.

In our series, various technical modifications were done to the Georgeson procedure. The baby is placed in the Trendlenberg position with legs widely split (frog's position / lithotomy), so that the position of the baby remains unchanged. We place a transcutaneously bladder stitch to overcome hindrance of vision even by the decompressed bladder. Traction on the undivided fistula helps in defining the sling shot of puborectalis and blunt dissection in the center of it for the pull through channel. Fistula ligation, as proposed, was omitted as the outcome was similar with just division. This avoids one of the most difficult steps in the constrained space. Conventional diathermy in a low setting current could be used just as effectively as a laparoscopic muscle stimulator for stimulating the puborectalis. Instead of a low profile step Verees needle with expanding sheath placed through proposed anal site for accurate creation of pull through channel, under suction canula guidance blunt dissection is done from the perineum and Hegar's dilators up to 12 mm size are successively railroaded to dilate the pull through channel. These modifications have made the procedure easier and saved time decreasing morbidity and early post operative recovery.

## CONCLUSIONS

LAARP provides excellent visualization of the rectal fistula and surrounding structures. In our experience, dividing the fistula without ligation is safe. LAARP allows accurate placement of the bowel through the anatomical midline and levator sling. Early postoperative recovery, early ambulation and decreased pain to the patient are seen in LAARP patients. Repair of associated defect at operation (i.e., hernia, identification and repair of cryptorchid testes) is possible. It is minimally invasive and leaves small abdominal and perineal wounds. We have found that LAARP is an alternative and more effective technique for high ARM over conventional methods. Earlier appearance and higher incidence of recto anal relaxation reflex is noted in LAARP patients.[9] Long term follow-up is essential for evaluation of final results.
